# Alcohol Involvement in Opioid Pain Reliever and Benzodiazepine Drug Abuse–Related Emergency Department Visits and Drug-Related Deaths — United States, 2010

**Published:** 2014-10-10

**Authors:** Christopher M. Jones, Leonard J. Paulozzi, Karin A. Mack

**Affiliations:** 1 Office of Public Health Strategy and Analysis, Office of the Commissioner, Food and Drug Administration; 2 Division of Unintentional Injury Prevention, National Center for Injury Prevention and Control, CDC; 3 Division of Analysis, Research, and Practice Integration, National Center for Injury Prevention and Control, CDC.

The abuse of prescription drugs has led to a significant increase in emergency department (ED) visits and drug-related deaths over the past decade. Opioid pain relievers (OPRs) and benzodiazepines are the prescription drugs most commonly involved in these events (1,2). Excessive alcohol consumption also accounts for a significant health burden and is common among groups that report high rates of prescription drug abuse (1,3,4). When taken with OPRs or benzodiazepines, alcohol increases central nervous system depression and the risk for overdose (5). Data describing alcohol involvement in OPR or benzodiazepine abuse are limited. To quantify alcohol involvement in OPR and benzodiazepine abuse and drug-related deaths and to inform prevention efforts, the Food and Drug Administration (FDA) and CDC analyzed 2010 data for drug abuse–related ED visits in the United States and drug-related deaths that involved OPRs and alcohol or benzodiazepines and alcohol in 13 states. The analyses showed alcohol was involved in 18.5% of OPR and 27.2% of benzodiazepine drug abuse-related ED visits and 22.1% of OPR and 21.4% of benzodiazepine drug-related deaths. These findings indicate that alcohol plays a significant role in OPR and benzodiazepine abuse. Interventions to reduce the abuse of alcohol and these drugs alone and in combination are needed.

The Substance Abuse and Mental Health Services Administration’s Drug Abuse Warning Network (DAWN) has been used to track the impact of drug use in the United States by monitoring hospital ED visits (DAWN ED) and drug-related deaths (DAWN ME) (1,6). DAWN collects data on illegal drugs, prescription and over-the-counter medications, and dietary supplements. In addition, DAWN collects information on alcohol involvement in these events. To be included in the DAWN database, the drug use (including alcohol) must be involved in the ED visit or death. Only drugs that are determined to be involved are recorded in the DAWN system. Unrelated drugs that are simply present are not recorded.

This report uses data from the 2010 DAWN ED public use file.* To estimate national ED visits, data were collected from a stratified, simple random sample of nonfederal, short-stay, general hospitals that operate 24-hour EDs. Poststratified weights were applied to the data from participating hospitals. This analysis included data from 237 hospitals on ED visits associated with drug misuse or abuse (referred to as abuse in this report), which is defined by DAWN ED as the group of ED visits that involve illicit drugs, alcohol-related visits (alcohol in combination with other drugs or alcohol alone for persons aged <21 years), and nonmedical use of pharmaceuticals (1). Nonmedical use is defined as taking more than prescribed, use without a prescription, taking a drug prescribed for someone else, malicious poisoning, and documented substance abuse involving pharmaceuticals. ED visits for suicide attempts and detoxification are included in the abuse category if illicit drugs are involved. Cases included those drug abuse–related ED visits that involved alcohol and OPRs or alcohol and benzodiazepines, whether alone or in combination with other drugs. ED visits involving more than one type of drug were counted in multiple categories. Estimates were suppressed if the relative standard error was >50% or if the estimate was based on fewer than 30 cases.

To complement the national ED visit data, 2010 data on deaths from DAWN ME from 13 states provided to CDC by SAMHSA also were used. In 2010, DAWN ME collected information on drug-related deaths referred to medical examiners and coroners (ME/Cs) in 373 counties in 157 metropolitan areas and 450 counties in 13 states. Data included in this analysis come from the 13 states that submitted data to DAWN ME (Delaware, Maine, Maryland, Massachusetts, New Hampshire, New Mexico, Oklahoma, Oregon, Rhode Island, Utah, Vermont, Virginia, and West Virginia). Cases were identified through a retrospective review of decedent case files. For this analysis, a case was any death determined by the ME/C to be related to drug use in which alcohol and OPRs or alcohol and benzodiazepines were involved, whether alone or in combination with other drugs. The drug use might have been for legitimate, therapeutic purposes or for the purpose of drug abuse or misuse. Per standard DAWN ME suppression rules, counts of deaths that were less than four but greater than zero were suppressed (6).

Percentages of drug abuse–related ED visits and drug-related deaths that involved alcohol were calculated for all OPRs and benzodiazepines as well as specific OPRs (fentanyl, hydrocodone, hydromorphone, methadone, oxycodone, and tramadol) and benzodiazepines (alprazolam, clonazepam, diazepam, and lorazepam), by age group, and by sex (for ED visits only). Percentages of drug abuse-related ED visits and drug-related deaths were calculated for both any ED visit or death that involved alcohol and OPRs, or alcohol and benzodiazepines, and for those ED visits and deaths where OPRs or benzodiazepines were the only drug class combined with alcohol. Because of public file formatting, age groups differed for ED visits and deaths. Sex was not available for drug-related deaths in the DAWN ME data file provided to CDC by SAMHSA. Differences in ED visits among various OPRs and benzodiazepines were tested using two-sided t tests. Risk ratios and associated 95% confidence intervals (CIs) were calculated to compare drug-related deaths caused by various OPRs and benzodiazepines.

Based on DAWN ED estimates, in 2010 in the United States, there were 438,718 ED visits related to OPR abuse and 408,021 ED visits related to benzodiazepine abuse, alone or in combination with other drugs. Of the OPR ED visits, an estimated 81,365 (18.5%) involved alcohol; of the benzodiazepine ED visits, 111,165 (27.2%) involved alcohol (Table 1). When restricted to ED visits where OPRs or benzodiazepines were the only drug classes involved, alcohol was involved in 26,446 (13.8%) OPR visits and 38,244 (34.1%) benzodiazepine visits.

Of the 3,883 OPR deaths in the 13-state DAWN ME data in 2010, 860 (22.1%) involved alcohol. For benzodiazepines, 324 (21.4%) of the 1,512 deaths involved alcohol (Table 2). Among single-drug class deaths, 393 (26.1%) OPR and 44 (72.1%) benzodiazepine deaths involved alcohol. OPRs stronger than hydrocodone, such as fentanyl, methadone, and hydromorphone, tended to have less alcohol involvement for both ED visits and deaths.

In 2010, the percentage of ED visits that involved OPRs and alcohol was highest among persons aged 30–44 years (20.6%) and 45–54 years (20.0%) (Figure). For benzodiazepine ED visits, the percentage was highest among persons aged 45–54 years (31.1%). ED visits involving alcohol and OPRs or alcohol and benzodiazepines were significantly more common among men than women: 22.9% (CI = 18.7%–27.7%) for men for OPRs compared with 13.5% (CI = 11.1%–16.4%) for women and 30.6% (CI = 26.7%–34.8%) for men for benzodiazepines compared with 24.1% (CI = 19.6%–29.2%) for women.

Among OPR deaths, persons aged 40–49 years (25.2%) and 50–59 years (25.3%) had the highest percentage of alcohol involvement. For benzodiazepine-related deaths, the highest percentage (27.7%) was among persons aged ≥60 years (Figure).

## Discussion

Alcohol was commonly involved in ED visits resulting from the abuse of OPRs or benzodiazepines as well as in deaths related to these drugs. Nearly one fifth of OPR abuse–related ED visits and more than one fourth of benzodiazepine abuse–related ED visits involved alcohol. Slightly more than one fifth of drug related deaths involved OPRs and alcohol and the same proportion applied to benzodiazepines and alcohol. Alcohol was more likely to be involved in single-drug class ED visits and deaths involving benzodiazepines compared with OPRs.

Alcohol involvement was higher in single-drug class ED visits for benzodiazepines compared with all ED visits involving benzodiazepines as well as single drug-class deaths for both OPRs and benzodiazepines compared with all deaths involving these drugs; this was especially pronounced for the benzodiazepine single-drug class deaths, for which 72.1% involved alcohol. This finding is consistent with the well characterized increase in central nervous system depression and overdose risk that results when alcohol is combined with these types of substances (5). It also indicates that benzodiazepines and weaker OPRs are less likely to cause such events without the additive effect of alcohol.

The percentage of alcohol involvement in ED visits for both OPRs and benzodiazepines was higher for men compared with women. Men report higher prevalence, frequency, and intensity of binge drinking compared with women, and this might have contributed to the higher percentage of alcohol involvement in ED visits among men seen in this study (3).

The results of the FDA and CDC analysis are consistent with previous reports. In West Virginia in 2006, 17.3% of unintentional pharmaceutical overdose deaths had alcohol as a contributing factor (7). In the National (Nationwide) Inpatient Sample, the largest publicly available all-payer inpatient health care database in the United States,† among persons aged 18–24 years, alcohol overdose was present in 20% of overdoses of opioids and related narcotics. Men had significantly higher rates: 25% compared with 15% for women, and were more likely to be hospitalized for overdoses combining opioids and alcohol. The nationwide study also found that the percentage of overdoses combining alcohol and drugs was higher among persons aged ≥25 years compared with those aged 18–24 years (8).

The findings in this report are subject to at least six limitations. First, the drugs (including alcohol) involved in ED visits and deaths might not all have been identified and documented. Second, distinguishing drugs taken for nonmedical and medical reasons is not always possible, especially when multiple drugs are involved. Third, DAWN ME does not rely on a statistical sampling of ME/Cs; findings cannot be considered representative of ME/Cs who did not participate, and results from the 13 states cannot be extrapolated to the entire United States. Fourth, state laws dictate which deaths are subject to ME/C review, and these laws vary by state. Fifth, toxicology testing practices vary depending on local concerns, funding, and testing technology, which affects the number of deaths determined to be DAWN ME cases and the number of deaths attributed to particular drugs. Finally, it was not possible to ascertain the amount of alcohol consumed, which limited the ability to look at outcomes by alcohol consumption level.

The fact that approximately one fifth of OPR drug abuse–related ED visits and drug-related deaths involve alcohol suggests the need for stronger prevention measures to mitigate this significant public health problem. OPRs and benzodiazepines are prescribed and dispensed by health care providers, and this presents an opportunity to discuss their risks, especially the serious risk of central nervous system depression when combined with alcohol or other depressants. However, only 16% of adults in the United States have discussed alcohol consumption with a health professional (9), and the percentage discussing other substance use is unknown. Interventions such as combined prevention programs that target alcohol and prescription drug abuse, systematic provider and patient education, and integration of screening and intervention services into the primary care health system to enable early identification of problematic alcohol and drug use might reduce the number of ED visits and deaths related to drug abuse and alcohol.

What is already known on this topic?

Opioid pain reliever and benzodiazepine abuse–related emergency department (ED) visits and drug-related deaths have increased significantly in the past decade. There is limited information on how often alcohol was involved in these events.

What is added by this report?

Based on data from a sample of EDs participating in the Drug Abuse Warning Network, alcohol was involved in an estimated 18.5% of opioid-related ED visits and 27.2% of benzodiazepine-related ED visits in the United States in 2010. The same year, based on medical examiner and coroner data from 13 states participating the Drug Abuse Warning Network, alcohol was involved in 22.1% of opioid-related deaths and 21.4% of benzodiazepine-related deaths. Compared with opioid pain relievers, alcohol was more likely to be involved in benzodiazepine ED visits (34.1% versus 13.8%) or deaths (72.1% versus 26.1%) when benzodiazepines were the only drugs involved.

What are the implications for public health practice?

Alcohol is involved in a significant proportion of opioid and benzodiazepine drug abuse–related ED visits and drug-related deaths. Interventions to educate health care providers and the public about the dangers of combining these substances need to be strengthened. Interventions that support early identification of and intervention in patients with alcohol and drug abuse problems should be integrated into the primary health care system.

## Figures and Tables

**FIGURE:**
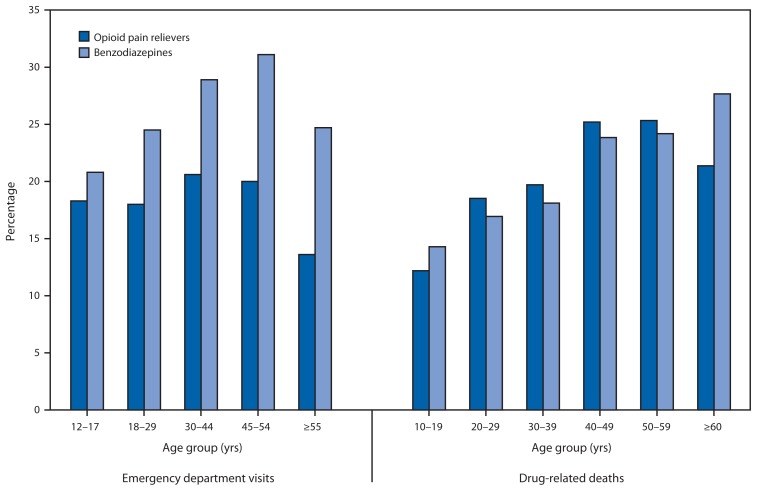
Percentage of opioid pain reliever and benzodiazepine drug abuse–related emergency department visits in the United States and drug-related deaths in 13 states that involved alcohol, by age group — Drug Abuse Warning Network, 2010

**TABLE 1:** Numbers and percentages of opioid pain reliever and benzodiazepine drug abuse–related emergency department visits that involved alcohol — United States, 2010

Alcohol and one or more drugs involved in emergency department visit			

	No.*	(%)*	(95% CI)
Opioid pain relievers	81,365	(18.5)	(15.3–22.3)
fentanyl/combinations	2,355	(10.2)†	(5.7–17.7)
hydrocodone/combinations	26,143	(22.6)	(19.8–25.7)
hydromorphone/combinations	2,619	(12.6)†	(7.7–20.0)
methadone	13,204	(17.3)	(9.4–29.8)
morphine/combinations	4,452	(13.1)†	(6.9–23.3)
oxycodone/combinations	35,878	(19.6)	(15.1–25.1)
tramadol/combinations	3,523	(19.7)	(12.5–29.6)
Benzodiazepines	111,165	(27.2)	(23.2–31.7)
alprazolam	39,573	(26.0)	(21.9–30.6)
clonazepam	22,089	(30.1)	(24.4–36.4)
diazepam	9,214	(28.8)	(21.7–37.2)
lorazepam	15,355	(34.6)	(25.7–44.6)
**Alcohol and single drug class involved in emergency department visit**			

	**No.** §	**(%)** §	**(95% CI)**

Opioid pain relievers	26,446	(13.8)	(10.4–18.0)
fentanyl/combinations	—¶	—	—
hydrocodone/combinations	7,251	(24.0)	(16.2–33.9)
hydromorphone/combinations	—	—	—
methadone	3,047	(11.9)†	(6.7–20.4)
morphine/combinations	396	(3.5)†	(1.5–8.0)
oxycodone/combinations	10,160	(15.9)	(9.9–24.5)
tramadol/combinations	818	(14.5)	(7.7–25.7)
Benzodiazepines	38,244	(34.1)	(29.5–39.0)
alprazolam	13,063	(31.4)	(25.6–37.9)
clonazepam	7,734	(33.6)	(27.1–40.9)
diazepam	2,622	(36.2)	(24.1–50.4)
lorazepam	5,207	(29.4)	(20.9–39.7)

**Abbreviation:** CI = confidence interval.

* Number and percentage of emergency department visits for abuse of drugs in one or more drug class that involved alcohol.

† Among opioid pain relievers, percentage is significantly (p<0.05) different from the percentge for hydrocodone/combinations. There were no statistically significant differences among benzodiazepines.

§ Number and percentage of emergency department visits for abuse of drugs in a single drug class that involved alcohol.

¶ Suppressed because of a relative standard error greater than 50% or an estimate based on fewer than 30 cases.

**TABLE 2:** Numbers and percentages of opioid pain reliever and benzodiazepine drug–related deaths that involved alcohol — 13 states, 2010

Alcohol and one or more drugs involved in emergency department visit				

	No.*	(%)*	RR	(95% CI)
Opioid pain relievers	860	(22.1)		
fentanyl/combinations	59	(17.0)	0.67	(0.51–0.87)†
hydrocodone/combinations	169	(25.5)	1.00	(referent)
hydromorphone/combinations	19	(23.8)	0.99	(0.62–1.41)
methadone	159	(16.3)	0.64	(0.53–0.78)†
morphine/combinations	161	(22.8)	0.90	(0.74–1.08)
oxycodone/combinations	304	(22.9)	0.90	(0.76–1.06)
tramadol/combinations	35	(15.9)	0.63	(0.45–0.87)†
Benzodiazepines	324	(21.4)		
alprazolam	145	(18.1)	1.00	(referent)
clonazepam	38	(18.9)	1.04	(0.76–1.44)
diazepam	85	(18.9)	1.04	(0.82–1.33)
lorazepam	21	(24.4)	1.35	(0.90–2.01)
**Alcohol and single drug class involved in emergency department visit**				

	**No.** §	**(%)** §	**RR**	**(95% CI)**

Opioid pain relievers	393	(26.1)		
fentanyl/combinations	19	(19.0)	0.44	(0.27–0.71)†
hydrocodone/combinations	30	(43.5)	1.00	(referent)
hydromorphone/combinations	4	(66.7)	1.53	(0.82–2.87)
methadone	61	(17.4)	0.40	(0.28–0.57)†
morphine/combinations	58	(31.7)	0.73	(0.52–1.03)
oxycodone/combinations	104	(35.3)	0.81	(0.59–1.11)
tramadol/combinations	4	(13.3)	0.31	(0.12–0.79)
Benzodiazepines	44	(72.1)		
alprazolam	18	(66.7)	1.00	(referent)
clonazepam	—¶	—	—	—
diazepam	8	(80.0)	1.20	(0.80–1.81)
lorazepam	0	(0)		

**Abbreviations:** RR = risk ratio; CI = confidence interval.

* Number and percentage of deaths from abuse of drugs in one or more drug class that involved alcohol.

† Among opioid pain relievers, percentage is significantly (p<0.05) different from the percentge for hydrocodone/combinations. There were no statistically significant differences among benzodiazepines.

§ Number and percentage of deaths from abuse of drugs in a single drug class that involved alcohol.

¶ Suppressed because death totals were greater than zero but less than four.

## Footnotes

* Additional information available at http://www.icpsr.umich.edu/icpsrweb/samhda.

† Additional information available at http://www.hcup-us.ahrq.gov/nisoverview.jsp.
